# Effectiveness of a health intervention based on WHO food safety manual in Iran

**DOI:** 10.1186/s12889-020-08541-8

**Published:** 2020-03-27

**Authors:** Mohtasham Ghaffari, Yadollah Mehrabi, Sakineh Rakhshanderou, Ali Safari-Moradabadi, Seyyede Zenab Jafarian

**Affiliations:** 1 Department of Public Health, School of Public Health and Safety, Shahid Beheshti University of Medical Sciences, Tehran, Iran; 2 Department of Epidemiology, School of Public Health and Safety, Shahid Beheshti University of Medical Sciences, Tehran, Iran; 3 Student Research Committee, School of Public Health and Safety, Shahid Beheshti University of Medical Sciences, Tehran, Iran; 4 School of Public Health and Safety, Shahid Beheshti University of Medical Sciences, Tehran, Iran

**Keywords:** WHO food safety manual, Intervention, Knowledge, Attitude, Behavior, Health volunteer

## Abstract

**Background:**

Food safety manual was developed by the World Health Organization (WHO) to train professionals to reduce the burden of foodborne diseases as a global strategy. The present pioneering research aimed to explore the effectiveness of an intervention based on the manual of five keys to safer food by WHO in enhancing the knowledge, attitude and behavior of Iranian Female Community Health Volunteers (FCHVs).

**Methods:**

In the present quasi-experimental research, FCHVs (*n* = 125) were selected and assigned to two groups, an intervention and a control. A modified version of the questionnaire based on WHO manual was used to measure knowledge, attitude and behavior of the sample. The questionnaire was first completed at the outset of the study (pre-test) and then once again in 2 months of the intervention (post-test). Face and content validity of the questionnaire was tested and confirmed. Cronbach’s alpha was used to test the reliability of the questionnaire along with the test-retest method of testing reliability. The data entered SPSS16 for statistical analysis. To this aim, Chi-squared test, dependent and independent samples T-test, ANOVA and ANCOVA were run. Partial population attributable risks were calculated and corresponding 95% confidence intervals (95% CIs) were estimated using a bootstrap method.

**Results:**

The two groups showed no statistically significant difference in the pretest (*p* > .05). In the post-test, the mean scores for all variables was higher in the intervention group than the control, and this difference between the two research groups was statistically significant (*p* < .001). When the volunteers were adjusted for age and experience in healthcare centers, the mean scores were significantly higher in the intervention group than the control (*p* < .001).

**Conclusion:**

It was revealed in the present study that the educational intervention based on five keys to food safety manual by WHO managed to improve participants’ knowledge, attitude and behavior. Translation of the target guideline in future can be a great help to researchers in prospective research.

**Trial Registration:**

**Retrospectively registered:** Iranian Registry of Clinical Trials IRCT20160822029485N4, at 2020-03-16.

## Background

The significance of maintaining human health and preventing diseases is known to all. Safe and healthy food is a key to prevention of diseases and protecting environment from germs. Annually, millions of people worldwide suffer from diseases transmittable through food. This has turned into a global issue [1]. Diseases that can be transmitted through food point to the prevalence of public health issues in developing and developed countries. Yet, health and economy in the former suffer more from these issues [2]. Human health often works through food safety frameworks and is more focused on proper diet, high-quality food and eating habits. Yet, it is noteworthy that socioeconomic issues can affect all procedures of food preparation from production to consumption. Thus, it can promote or damage individual and social health [3]. Food-related diseases are reported to prevail in food service providing areas including restaurants, hospitals, schools, and so on [4]. Food safety is a complicated matter of concern. Despite quite many campaigns on food safety and educational efforts along with decades of exploratory microbiology, food-related diseases are the main source of human diseases [5]. Reports of disease outbreaks spanning from 2006 to 2010 have shown an increase of about nine fold [6]. The majority of outbreaks and cases of diseases have been transmitted from contaminated food to human. Health authorities’ behaviors and methods have increased the rate of these diseases and can be related to particular organisms [7]. Therefore, a high percentage of diseases is caused by improper preparation of food materials at home [8]. Women produce between 60 and 80% of food materials in many of the developing countries and account for half of food products at a global scale [9].

The growing number of diseases transmittable through food has made all countries attempt extensively to improve food safety [10]. Some research revealed that people’s higher knowledge tremendously affects their nutrition [11]. Some other research revealed a correlation between food safety education and raising knowledge of food service providers [12]. Moreover, copious educational interventions have been run to develop ready-to-eat food. Yet, these interventions are only effective when they are evidence-based. WHO has recently developed five keys to food safety [13].

## Five keys to food safety promotion by WHO

WHO emphasized health promotion and food safety, consulted food safety experts and consumers at risk for a year and proposed a five-step guideline to prevent food-related diseases. At the core of the guideline there are five key points to consider: keeping clean, separating fresh from cooked food, full cooking, storing food at safe temperature and consume healthy drinking water and raw materials [14]. These five keys to safe food are of a great significance in developing countries. Those involved in food production in these countries can use this information to affect the safety of food materials. This guideline has been translated in more than 40 languages of the world and is currently used worldwide to convey the healthy message sent by WHO [14]. Along with the 5-key to food safety poster, WHO has published a general book of instructions which acts as a framework for teachers and other institutes interested in food safety to develop instructional materials and programs for those groups at risk [15]. The results of the Ghana confirmed that an educational intervention based on the 5 keys helps to raise the knowledge and improve the performance of salespeople [16].

In the present research, FCHVs entered the study to learn the content proposed by WHO about food safety.

Local women older than 25 years receiving an 18-day training of different Primary Health Cares (PHC) concerns are called FCHvs. The training is concerned with mother and child health care matters, mainly [17]. These women work part time, 5–6 h per week. This time may change contingent upon the programs FCHVs take part in. It seems that FCHVs are committed to volunteer because their retention rate is high (96%) [18]. The main reasons for high motivation are social respect, religious and moral duty, in policy making perspective [19], these volunteers have not had a voice in policy making yet. Also, they have not received any payment for their activities and offered services [20].

Local FCHV in Iran play a key role in primary healthcare provision and teaching the urban community. These people are the first and foremost means of communication between urban community and the health network. They lie at the core of urban community and always communicate with families. They play an effective role in raising the knowledge of women and mothers and changing their attitude. A body of related literature has proved the role of FCHV in raising the knowledge of society under cover [21–24]. Thus, it can be a proper target group to disseminate health-related information. A review of the relate literature showed that no relevant research has been conducted, so far, in Iran in relation to the five keys. Considering the key role of women in food safety and the role of FCHVs in educating women, the present research aimed to explore the effectiveness of a health intervention in the light of WHO five-keys in FCHVs’ knowledge, attitude and behavior.

## Methods

### Research domain and population

Damavand is a county in Tehran province and accommodates 125,480 residents. According to the latest national division reports, this county is divided into two districts, the center and Rudehen. These two districts together consist of 5 cities (Damavand, Rudehen, Absard, Abali and Kilan) and 111 villages. This county has got 5 healthcare centers, 1 health station and 1 hospital affiliated with Shahid Beheshti University of medical sciences. This county was selected in this research due to its geographical location, ethnic diversity, inclusion of urban and rural populations together and ease of access and coordination of team work from Shahid Beheshti University.

### Research design, sample selection, inclusion/exclusion criteria

The present quasi-experimental research was conducted as a census on all FCHVs in the target health centers. FCHVs of three centers were taken as the experiment group (*n* = 65) and the other two as the control (*n* = 60). The inclusion criteria were: work as health volunteer in the target health center, consent to participate in the research and active participation in educational program. The exclusion criterion was a volunteer’s unwillingness to continue with the research for any reason. Finally, 6 subjects from the experiment group and 10 from the control were excluded from the study due to absence from educational sessions or pre-test/post-test sessions.

### Instrumentation

To explore the effectiveness of the intervention, a questionnaire was developed in the light of WHO guideline.

#### A: Demographic information

In this section, participants’ age, work experience, education, marital status and previous experience of education on food safety.

#### B: WHO guideline

There were 31 items in the light of the 5 keys to food safety in WHO guideline. The knowledge sub-section was comprised of 11 items to be scored as 0 for a ‘false’, 1 as ‘don’t know’ and 2 as ‘true’ answer. The attitude sub-section consisted of 10 items to be rated on a Likert scale: 0 (disagree), 1 (not sure) and 2 (agree). As for behavior, there were 10 items involved to be rated as ‘always’, ‘often’, ‘sometimes’, ‘rarely’ and ‘never’. These items were to be rated between 1 and 5 [25].

### Modified and naturalized instrumentation

**Fig. 1:**
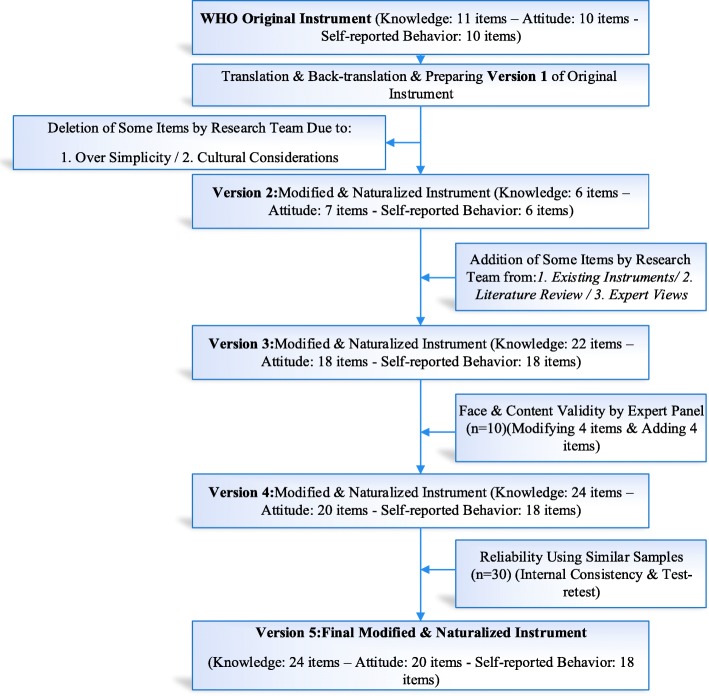
Instrument preparation process flowchart

When the main WHO guideline was translated, some items within the questionnaire were too simple and eliminated as they did not match the life-style of the target population. An instance was the insertion of a thermometer inside meat to adjust its temperature while cooking. Such items were to be omitted in naturalization according to WHO guideline. The face and content validity of the questionnaire was checked by a panel of experts (*n* = 10) in healthcare sciences and nutrition. Among the corrective changes made to the questionnaire adapting 4 items and adding 4 more. Finally, there were 62 items within this section of the questionnaire developed in the light of the 5 keys to food safety in WHO guideline. The *knowledge* sub-section was comprised of 24 items to be scored as 0 for a ‘false’, 1 as ‘don’t know’ and 2 as ‘true’ answer. Overall, a maximum score for knowledge was 48. The *attitude* sub-section consisted of 20 items to be rated on a Likert scale: 0 (disagree), 1 (not sure) and 2 (agree). The total attitude score ranged between 0 and 40. As for *behavior*, there were 18 items involved to be rated as ‘always’, ‘often’, ‘sometimes’, ‘rarely’ and ‘never’. These items were to be rated between 1 and 5. Several items were reversely score. The range of scores for the whole scale was 18–90. To test the reliability of items, the questionnaire was initially submitted to 30 female community health volunteer working in Shemiranat health centers (other than the participants of the research). To test the reliability of the *knowledge* sub-section, the test-retest method was used. Ten of the same FCHVs were asked in 2 weeks of the test (as a retest) to respond to the questionnaire. The reliability coefficient of *knowledge* was estimated at .79, and thus confirmed. To test the reliability of *attitude* and *behavior*, Cronbach’s test of internal consistency was run. The reliability coefficients of the two were respectively .76 and .92 (Fig. 1).

#### Intervention

The pretest was run using the questionnaire for both groups, experiment and control. This phase of the research was done to determine educational content, number of intervention sessions and teaching methods. The educational course was held for the experiment group in a class inside each health center in a city or health station for a full month. The control group did not receive any intervention by the researcher. After the end of the research period, the control group was receive the contents that were provided for training in the test group.

The content of the food safety guideline was translated by the present research team and complemented with the related literature. It is noteworthy that the food safety poster was already translated by the Food and Drug Organization. Yet, the guideline was accessed through WHO website and as investigated by the researcher was found to be untranslated by Food and Drug Organization. Thus, the present research team translated this guideline into Persian. The five-key guideline to safe and healthy food was developed in two parts. The first part presents the background and purpose of the guideline while the second part presents the five keys to provide access to healthier food. In the second part, the information content of the 5-key poster for a healthier diet is elaborated on and several suggestions are made on how to communicate the message. Besides, two posters illustrating food safety were put up in the FCHVs’ room to further encourage them and raise their knowledge. At the end of each session, a pamphlet was availed to them too.

The instructional content of each session was presented in accordance with learners’ learning power using reliable scientific sources and experts’ comments. To ensure of the correspondence between the educational content with participants’ level of understanding, this content was provided to 10 health volunteers similar to those in the main research, as a pilot test. The intervention ran for 1 month in five 90-min sessions in the form of lecture and PowerPoint presentation. Question and answer sessions were also held. Educational pamphlets were developed. Group discussions were held for the experiment group, and at the end of each session, a pamphlet was developed corresponding to the five keys (keeping food materials clean, separating raw and cooked foods, cook food thoroughly, store it at safe temperature, and drink healthy water and fresh food stuff).

Two months after the educational intervention, a posttest was run for the experiment and control groups.

### Ethical considerations

Participation in this research was voluntarily and willingly. All conduction procedures were authorized by the health center in Damavand. Before submitting the questionnaires to health volunteers, they were told about the purpose of research and were asked for an oral consent. They were ensured of the confidentiality of the information they provided. In the final phase of the research, the teaching content offered to the experiment group (including the guideline, pamphlet and posters) was also availed to the control group in an intensive session.

### Data analysis

The data entered SPSS16 for statistical analysis. To test the normality of data, KS test was run and the required histograms were drawn and checked. To compare the two research groups in terms of the target variables, before the intervention, chi-squared test, Mann-Whitney U-test and independent samples T-test were run. To test intra-group variation, dependent samples T-test was used. To test the effectiveness of the educational intervention in improving health volunteers’ knowledge, attitude and behavior in the experiment group, as compared to the control, adjustment for age and work experience was done and then ANCOVA was run. Partial population attributable risks were calculated and corresponding 95% confidence intervals (95% CIs) were estimated using a bootstrap method.

## Results

The majority of participants in both groups and in all phases were selected conveniently. Only 7 subjects (6 healthy subjects in the experiment group and 1 in the control) were excluded. As the researcher was present during the questionnaire completion phase, the collected data lacked any defective or incomplete answer.

In the present research, 109 health volunteers (59 in the experiment and 50 in the control group) participated with an average age of 44 years (min = 21 years, max = 63 years) and work experience of 10 years. As chi-square test results showed, there was no statistically significant difference between the two research groups before the intervention in terms of age and work experience (*p* = .937). Moreover, the two groups did not differ significantly in terms of marital status, education and previous experience of instructions on food safety, spouse’s education and occupation. Also Independent-samples T-test results revealed no statistically significant difference between knowledge, attitude and behavior scores of the two groups before the intervention (*p* > .05) (Table 1).

**Table 1:** Distribution of demographic features and another variables in FCHVs in the pretest

Variable	Sub-groups	Experiment Group F (%)	Control group F (%)	*P*-Value
Age	20–40 y	18(30.5)	14(28)	0.937
	41–55	36(61)	31(62)	
	> 55	5(8.5)	5(10)	
Education level	Elementary school, adult school	13(22)	14(28)	.166
	Junior high school	25(42.4)	13(26)	
	High school, diploma	16(27.1)	21(42)	
	University	5(8.5)	2(4)	
Marital status	Married	50(84.7)	44(88)	.623
	Single	9(15.3)	6(12)	
Previous experience of instructions on food safety	Yes	47(79.7)	42(84)	.56
	No	12(20.3)	8(16)	
		Mean ± SD	Mean ± SD	
Work experience (Year)		10.47 ± 7.28	10.06 ± 6.19	.814
		Mean Score ± SD	Mean Score ± SD	
knowledge		35.77 ± 5.8	36.06 ± 6.45	0.812
Attitude		32.38 ± 5.97	31.78 ± 6.75	0.618
Behavior		75.05 ± 17.39	73.50 ± 17.48	0.644

Effectiveness of the educational intervention in improving health volunteers’ knowledge, attitude and behavior, once age and work experience factors were adjusted, was checked through ANCOVA. The results revealed that the intervention was successful in improving participants’ knowledge, attitude and behavior significantly. Paired t-test analysis (With bootstrap method) also showed a significant difference between the mean score of knowledge (*P* = 0.001, 95%CI: 4.15–7.16), attitude (*P* = 0.001, 95%CI: 4.84–7.38) and behavior (*P* = 0.002, 95%CI: 6.49–15.38) before and after the educational intervention in the experimental group. No such a significant difference was observed in the control group (*P* > 0.05) (Table 2).

**Table 2:** Comparison of the two research groups in terms of knowledge, attitude and behavior before and after intervention

Variable	Group	MD^a^ ± SD	Bootstrap^b^			
			Bias	*P*-Value	95% Confidence Interval	
					Lower	Upper
knowledge	Experimental	5.62 ± 6.02	−.03	.001	4.15	7.16
	Control	.24 ± 3.96	.01	.651	−.81	1.32
Attitude	Experimental	6.03 ± 5.07	−.009	.001	4.84	7.38
	Control	.18 ± 3.58	.01	.745	−.82	1.21
Behavior	Experimental	10.89 ± 10.16	−.08	.002	6.49	15.38
	Control	6.44 ± 9.21	−.06	.034	3.12	10.32

^a^Mean score difference, ^b^unless otherwise noted, bootstrap results are based on 1000 bootstrap samples

As the results pair test (with bootstrap method) showed, statistically significant differences were observed in the experiment group in terms of the five keys to food safety (Table 3).

**Table 3:** Comparison of mean score variables based on WHO food safety 5-keys before and after intervention in experiment and control groups

***Variable***	Sub variable^a^	Group	MD^b^ ± SD	Bootstrap^c^			
				Bias	*P*-Value	95% Confidence Interval	
						Lower	Upper
*Knowledge*	Key 1	Experimental	1.05 ± 1.75	−.01	.001	.61	1.49
		Control	−.42 ± 1.38	−.002	.048	−.85	−.06
	Key 2	Experimental	.76 ± 1.45	.002	.001	.40	1.15
		Control	−.08 ± 1.3	.007	.654	−.42	.28
	Key 3	Experimental	1.15 ± 1.63	.00	.001	.76	1.59
		Control	.68 ± 0.86	−.005	.001	.42	.92
	Key 4	Experimental	.25 ± 1.58	.016	.247	−.15	.66
		Control	−.26 ± 1.08	−.003	.091	−.56	.02
	Key 5	Experimental	2.37 ± 2.65	.003	.001	1.71	3.08
		Control	.32 ± 1.62	−.009	.155	−.15	.70
Attitude	Key 1	Experimental	1.38 ± 1.93	−.005	.001	.91	1.91
		Control	.24 ± 1.43	−.004	.263	−.15	.64
	Key 2	Experimental	.38 ± 1.2	.002	.016	.10	.72
		Control	−.40 ± 1.06	−.003	.016	−.70	−.14
	Key 3	Experimental	.28 ± 0.9	.002	.029	.06	.52
		Control	.00 ± 0.63	.000	1.000	−.18	.18
	Key 4	Experimental	.54 ± 1.3	.002	.008	.23	.89
		Control	.04 ± 0.83	.001	.738	−.17	.28
	Key 5	Experimental	3.42 ± 2.21	−.001	.001	2.86	4.05
		Control	.30 ± 1.74	−.008	.230	−.16	.77
Behavior	Key 1	Experimental	1.94 ± 5.73	.03	.019	.55	3.60
		Control	1.08 ± 3.99	.013	.092	.14	2.36
	Key 2	Experimental	1.86 ± 3.52	.006	.002	1.06	2.81
		Control	1.38 ± 2.96	.021	.017	.66	2.25
	Key 3	Experimental	1.81 ± 2.34	.01405	.001	1.25424	2.50847
		Control	1.36 ± 2.36	.016	.002	.76	2.01
	Key 4	Experimental	2.44 ± 2.65	.007	.001	1.76	3.15
		Control	.98 ± 2.26	.006	.010	.40	1.67
	Key 5	Experimental	2.83 ± 6.45	.03	.008	1.33	4.61
		Control	1.64 ± 5.1	.01	.081	.40	3.23

^a^K1: keep clean, K2; Separate raw and cooked K3: Cook thoroughly, K4: Keep food at safe temperatures, K5: Use safe water and raw materials, ^b^Mean score difference, ^c^unless otherwise noted, bootstrap results are based on 1000 bootstrap samples

## Discussion

The present research aimed to explore the effectiveness of an educational program based on a food safety guideline proposed by WHO in promoting health volunteers’ knowledge, attitude and behavior. In an extensive review of the related literature, no similar research was found in Iran based on the five keys to food safety proposed by WHO.

The present results confirmed the effectiveness of the educational intervention among the participants. In a similar investigation by “Donkor et al 2009” on street salesmen based on the five keys proposed by WHO, the intervention managed to raise the knowledge of 67.6% of participants [16]. Moreover, the research conducted by “Yarrow et al 2009” on university students showed that the educational intervention could increase the mean knowledge score of food safety [26]. Some other research by “Mullan et al 2010” indicated that an educational intervention based on the theory of planned behavior (TPB) could help to increase knowledge of food safety among 17–46 year-old Australian population [27]. In Iran, this research indicated the effectiveness of educational intervention in raising knowledge of food safety [23, 28, 29]. Raising knowledge of the safety of highly consumed food material is essential but is currently insufficient for food consumers. As reported by Acikel et al., knowledge plays a key role in improving the safety methods used by authorities in food industry [30]. Comparatively, quite many investigations pointed out the fact that knowledge alone cannot improve the food safety approaches of food health authorities [31–33]. Cuprasitrut et al. also pointed out the essentiality of food instructions especially on how to store it [34].

As the independent-samples T-test results showed, the two research groups were significantly different in terms of the attitude score after intervention. The mean attitude score was significantly increased in the experiment group after intervention as compared to the control. Attitude reflects traditional beliefs and can be a barrier to the adoption of the right approach. A positive attitude can improve endeavors and the opposite way [35]. In the present research, positive changes were made to participants’ attitude toward safety. It seems that teaching the content of food safety guideline proposed by WHO based on five behavioral constructs was effective in creating a positive attitude in health volunteers. In their research, “Yellow et al. 2009” showed that the educational intervention managed to improve university students’ attitude [26]. In some other research, “Burke & Dworkin 2013” showed that educational intervention effectively influenced the attitude of patients afflicted with AIDS toward food safety [36]. Pirsaheb et al. also showed that the mean attitude score of those in charge of food quality control was increased after the educational intervention [29]. These results were consistent with those of the present research.

It was found in this research that the mean behavior scores of the experiment and control groups diverged significantly in 2 months of intervention. This finding was similar to the findings reported by “Donkor et al 2009”, Yarrow et al. and Burke et al. [16, 26, 36]. In some other research on patients afflicted with AIDS in 2013, Dworkin et al. indicated that 85% of patients changed their behavior concerning food safety after receiving and reading the booklets [37]. In the Iranian context, the research findings by Pirsaheb et al. indicated the improved performance of food authorities after the educational intervention [29]. The research findings reported by Mullan et al. we’re not consistent with the present findings, as their educational intervention did not manage to improve participants’ behavior [27]. In their report, York et al. maintained that food servants received a higher score of food safety knowledge after the education course [38]. Other investigations showed that knowledge of food health and safety does not necessarily translate into behavior [39–42]. Worsfold et al. pinpointed that change of behavior to access healthy food stuff can occur when the acquired knowledge and skills are persistently practiced and used [43]. Constant education and support of management are among the key elements in transferring knowledge to behavior [44]. Therefore, there is a need for further research to understand factors that limit knowledge transfer to healthy food practice among those in charge. Thus, cutting down on theoretical concepts used in education should be investigated [45]. There have been several suggestions made with this concern that can help or can maximize intervention strategies: 1) educating food providers at workplace to improve their perception of methods or approaches. Moreover, at the workplace, the presence of a teacher or leader can enhance theoretical ideas in practice [46, 47]. 2) Managers and supervisors can be effectively included in interventions relevant to the right forms of performance. A knowledgeable supervisor or monitor can help to correct inappropriate approaches [48, 49]. 3) an appropriate environment that is well-equipped is needed to implement proper production methods [50, 51], 4) Such motivating factors as motivation and self-efficacy can play a key role in promoting education and welcoming required measures [49].

There are some positive points to this study. This study is in fact a pioneering interventional research in the light of WHO food safety manual conducted in Iran. It presented the Persian translated version of the manual which included a naturalization and modification procedure to prepare a proper evaluation instrument for knowledge, attitude and behavior. This study further enjoyed a focus on women as a crucial sub-group in producing food stuff in many developing countries including Iran as well as the world. It also focused on health volunteers in particular so as to multiply the value of the intervention. Also, there were several limitations in the present research including the high age of most of health volunteers. The use of self-reports and the large number of items within the questionnaire could also cause fatigue and lower precision in answers.

## Conclusion

It was revealed in the present study that the educational intervention based on five keys to food safety manual by WHO managed to improve participants’ knowledge, attitude and behavior. Translation of the target guideline in future can be a great help to researchers in prospective research. It is also essential to consider women’s role in family as the target group for education and finally hold instructional sessions based on the food safety principles proposed by WHO for those involved in healthcare provision, especially on food safety. One issue that needs to be dealt with is the limited sample size in the present research, which is explained by the demographic-geographical conditions of the target population. In the present research, all FCHVs were included as a census. Yet, due to the research design (dividing the sample to a control and a treatment group), the sample size seems small. Yet, specialized tests can be used in the present research to prove that similar results can be obtained from larger samples. Here, the effectiveness of the educational interventions was confirmed considering the number of sessions as well as the educational content related to the target group in raising knowledge, improving attitude and nutritional safety-related behavior. Thus, there are chances that similar studies at a larger scale, with more sessions included and different methodological procedures (use of local media in studies of larger scale vs. the use of PowerPoint presentation and training courses in the present research) yield results consistent with the present findings. In addition, conducting longitudinal studies as further research are suggested to explore the effects of such interventions on public health issues. Besides, further research is required to explore outcome indices (e.g. the rate of food poisoning with diseases transmitted through foods caused by low nutritional safety).

## Data Availability

The datasets used and analyzed during the current study are available from the corresponding author on reasonable request.

## Abbreviations

FCHVs: Female Community Health Volunteers
PHC: Primary Health Cares
WHO: World Health Organization
